# Linking Gait Dynamics to Mechanical Cost of Legged Locomotion

**DOI:** 10.3389/frobt.2018.00111

**Published:** 2018-10-17

**Authors:** David V. Lee, Sarah L. Harris

**Affiliations:** ^1^School of Life Sciences, University of Nevada Las Vegas, Las Vegas, NV, United States; ^2^Department of Electrical and Computer Engineering, University of Nevada Las Vegas, Las Vegas, NV, United States

**Keywords:** biomechanics, energetics, economy, walking, running, bipedal, quadrupedal, comparative

## Abstract

For millenia, legged locomotion has been of central importance to humans for hunting, agriculture, transportation, sport, and warfare. Today, the same principal considerations of locomotor performance and economy apply to legged systems designed to serve, assist, or be worn by humans in urban and natural environments. Energy comes at a premium not only for animals, wherein suitably fast and economical gaits are selected through organic evolution, but also for legged robots that must carry sufficient energy in their batteries. Although a robot's energy is spent at many levels, from control systems to actuators, we suggest that the mechanical cost of transport is an integral energy expenditure for any legged system—and measuring this cost permits the most direct comparison between gaits of legged animals and robots. Although legged robots have matched or even improved upon total cost of transport of animals, this is typically achieved by choosing extremely slow speeds or by using regenerative mechanisms. Legged robots have not yet reached the low mechanical cost of transport achieved at speeds used by bipedal and quadrupedal animals. Here we consider approaches used to analyze gaits and discuss a framework, termed mechanical cost analysis, that can be used to evaluate the economy of legged systems. This method uses a point mass perspective to evaluate the entire stride as well as to identify individual events that accrue mechanical cost. The analysis of gait began at the turn of the last century with spatiotemporal analysis facilitated by the advent of cine film. These advances gave rise to the “gait diagram,” which plots duty factors and phase separations between footfalls. This approach was supplanted in the following decades by methods using force platforms to determine forces and motions of the center of mass (CoM)—and analytical models that characterize gait according to fluctuations in potential and kinetic energy. Mechanical cost analysis draws from these approaches and provides a unified framework that interprets the spatiotemporal sequencing of leg contacts within the context of CoM dynamics to determine mechanical cost in every instance of the stride. Diverse gaits can be evaluated and compared in biological and engineered systems using mechanical cost analysis.

## Introduction

As with any structural or functional animal feature, it is important to consider locomotion through the lens of organic evolution. In nature, the process of natural selection replaces the forward design used in engineering. As emphasized by Vogel (2013), instead of being optimized through a design process, biological designs emerge to be just good enough so that the organism can survive, compete, and reproduce in its ecological niche. In addition, natural selection is constrained by phylogenetic, constructional, and developmental factors. Leg number (Shubin et al., 1997), the composition of the skeleton (Currey, 2002) or cuticle (Hopkins and Kramer, 1992), as well as muscles and their innervations (Diogo and Abdala, 2010) are generally conserved within taxa. For example, when considering leg number, true crabs (Decapoda) have ten legs, insects (Hexapoda) have six, and terrestrial vertebrates (Tetrapoda) have four (except when lost in certain lineages). Crabs and insects (Arthropoda) also have an exoskeleton made of a chitin composite, whereas vertebrates have an endoskeleton made of bone. Such fundamental features are conserved within a given phylogenetic group and therefore place limits on the mechanical solutions that might emerge. Phylogenetic, constructional, and developmental features of an animal's biology, as well as body size, lifespan, and other characteristics, impose severe constraints on the structural and functional “design solutions” available. As extreme examples, biological constraints make it so that we cannot replace our bones with a jointed exoskeleton or develop longer sarcomeres for greater force production. Hence, the locomotion and neuromusculoskeletal function observed in any species should not be seen as “optimal” but simply as a competent solution emerging from the highly constrained process of natural selection. Here we review key features of nature's strategies for terrestrial locomotion, focusing on gait and center of mass dynamics with a view toward informing the design of legged robots.

## GAIT

Locomotion is fundamental for foraging, prey capture, predator evasion, securing territory, finding mates, and migration. Animals use various modes of locomotion called gaits as defined by their movement patterns. Gait is conventionally defined by temporal footfall patterns and, for terrestrial animals, the footfall sequence is the primary identifier of gait. Footfall sequence is quantified by the phase relationships of individual legs, expressing the time of foot contact as a fraction or percentage of stride period. For example, for walking, one foot lands at the beginning of the stride (0%) and the second foot lands at mid-stride (50%).

**Figure 1:**
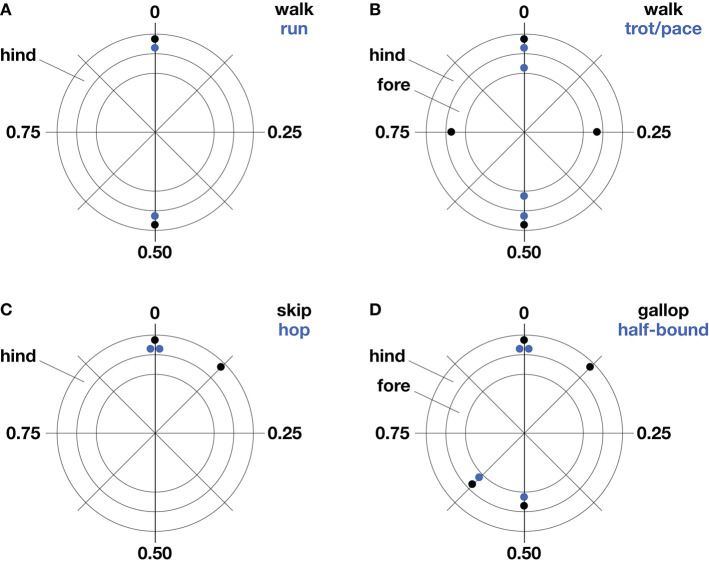
Common gaits of bipeds **(A,C)** and quadrupeds **(B,D)**. Stereotypical foot contact phases are represented as a fraction of stride period on polar plots. The outer ring represents hind limb contacts and the inner ring, forelimb contacts. Forelimb and hind limb pairs one-half cycle out phase indicate symmetrical gait **(A,B)** while substantial deviations from this phase relationship in either pair indicate asymmetrical gait **(C,D)**.

Yet a phase-based definition of gait is often incomplete and can fail to distinguish different gaits because they may have similar or even identical phase relationships. For example, bipedal walking and running show the same left-right-left sequencing of footfalls, with phases of initial contact of alternating feet at 0% and 50% of the stride period (Figure 1A). Hence, the distinction between walking and running traditionally relies upon the duty factor, which represents the duration of a given footfall as a fraction of stride period. On this basis, human running is distinguished from walking by its duty factor of <0.5, which specifies an aerial period in the case of bipedal running—but not necessarily in quadrupedal or multilegged running, where footfalls with duty factors <0.5 may be sequenced such that there is no aerial phase. In contrast to humans, birds are bipedal striders that blur the distinction between walking and running. Using duty factors >0.5, they exhibit exhibit “grounded” running without an aerial phase. But how do we know that grounded running is indeed running as opposed to walking? Despite the convenience of separating running from walking on the basis of duty factor, it is clear that criteria beyond temporal footfall metrics are needed to distinguish the underlying physics of gait. This section focuses on the broad utility of temporal patterns in the definition of gait, and section Center of Mass Dynamics addresses similarities and differences in the underlying dynamics of various gaits.

### Symmetrical vs. asymmetrical gaits

Legged gaits are broadly characterized as symmetrical or asymmetrical according to the phase relationships of left-right pairs of legs. If the left and right legs of a pair are one-half stride cycle out of phase with one another, the gait is defined as symmetrical—if not, the gait is asymmetrical. This convention spans animals of different leg number. For example, bipedal running of humans, quadrupedal trotting of dogs, pacing of camels, and hexapedal trotting of cockroaches are all symmetrical gaits because left-right pairs of fore-, mid-, and hind-legs are one-half cycle out of phase with each other. Regardless of leg number, gait symmetry is defined by this half-cycle phase relationship of the left and right legs at a given cranio-caudal position.

The number of legs limits the number of leg sequencing options, such that gait possibilities for bipeds are restricted to symmetrical striding gaits (walking and running) and asymmetrical hopping and skipping. Quadrupeds use five symmetrical gaits (lateral and diagonal sequence walking, trotting, pacing, and ambling) and six asymmetrical gaits (lope, transverse and rotary gallops, half-bound, bound, and pronk). These are broad definitions, and it is important to note that phase separations between foot contacts show substantial variation within gaits, as illustrated in (Hildebrand, 1965, 1968) plots for the gaits of horses and dogs.

### Bipedal gaits

Bipedal striding gaits, including our own walking and running, are symmetrical by definition (Figure 1A). These gaits are used by birds and historically by theropod dinosaurs, which comprise the greatest diversity of bipedal striders. Humans and birds (except small songbirds) walk at slow speeds and run at fast speeds (Small songbirds typically use hopping rather than striding gaits). Some great apes and monkeys are facultative bipeds, occasionally walking or running for short distances. Lizards and cockroaches may run bipedally at their fastest speeds by pitching their body into a more upright attitude and straightening their legs as they transition from a quadrupedal (Irschick and Jayne, 1999) or hexapedal trot (Full and Tu, 1991). This seemingly odd behavior increases speed by increasing stride length. In general, bipedal striders achieve greater absolute stride lengths than quadrupeds of the same body mass (Reynolds, 1987), and this has been argued to be an advantage for endurance runners of our own genus—for example, in persistence hunting of quadrupeds or aggressive scavenging in competition with quadrupeds (Carrier, 1984; Bramble and Lieberman, 2004).

Although the presence of only two legs allows relatively few sequencing options, two asymmetrical gaits—hopping and skipping—are used by bipeds (Figure 1C). Bipedal hopping gaits are common in mammals and birds, particularly at small body size. It may seem counterintuitive that the paired contacts of right and left hind limbs should indicate an asymmetrical gait, however, this deviates from the symmetry criterion that right and left contacts be one-half stride cycle out of phase. Bipedal hopping is found convergently in several groups of rodents, including springhares, kangaroo rats, and jerboas—and the only large bipedal hoppers are the wallabies and kangaroos, with male red kangaroos equaling human body mass (reviewed by McGowan and Collins, 2018). Bipedal skipping, a gait occasionally used by children and sometimes in reduced-gravity conditions, is the only asymmetrical gait used by humans (Minetti, 1998, 2001a,b; Ackermann and van den Bogert, 2012). Monkeys and apes with forelimbs specialized for brachiation in arboreal environments sometimes use side-ways bipedal skipping gaits on the ground, and this is typified by the fast, dance-like gait of the gibbon/siamang (Vereecke et al., 2006). Bipedal skipping gaits are thought to be akin to the asymmetrical galloping of quadrupeds and this is supported by similarities in relative phase of the hindlegs (Figures 1C,D), as well as by the analysis of CoM dynamics (Minetti, 1998).

### Quadrupedal gaits

The symmetrical gaits of quadrupeds include the walk, trot, pace, and amble. Quadrupedal mammals, crocodiles, lizards, and salamanders normally walk at slow speeds and trot at intermediate speeds, while the pace and amble are used by just a few mammals. During walking, leg contact phases are typically separated by about 25% and the order of contact is typically a hind foot, followed by the ipsilateral forefoot, followed by the contralateral hind foot and forefoot (Figure 1B). This most commonly used sequence of footfalls is called a lateral sequence walk. Yet most primates walk with a diagonal sequence, which is thought to provide better roll stability when walking on branches (Hildebrand, 1980; Cartmill et al., 2002). When walking on branches, however, this advantage of diagonal support may be diminished by a relatively narrow stance compared to that of terrestrial locomotion. A full mechanistic understanding of this difference between primates and typical quadrupeds will likely require consideration of individual foot force and torque in addition to spatiotemporal considerations (e.g., Shapiro and Raichlen, 2005, 2007; Cartmill et al., 2007). Notwithstanding these distinctions, simple models have shown that the lateral sequence walking gait used by most quadrupeds provides a stable tripod of support, termed “static stability” (McGhee, 1976; Alexander, 1984), and a mathematical model of slow walking found this to be the most stable of the three possible gaits that can provide a continuous tripod of support (McGhee and Frank, 1968), while the other two solutions appear not be used by animals.

Quadrupeds use the remaining symmetrical gaits—the trot, pace, and amble—at intermediate speeds. Trotting is the symmetrical gait most widely used at intermediate speeds and its relative phases resemble those of bipedal striding (Figure 1B). During trotting, diagonal legs are in phase, providing a supporting leg on each side to resist rolling that may be induced by a sprawled posture. In contrast, ipsilateral legs are in phase during pacing, discarding roll stabilization but avoiding fore-hind interference in mammals with especially long legs, such as camels, as well as some dogs and horses (Hildebrand, 1968). The transition between walking and trotting occurs at a similar relative speed for all quadrupeds. However, the top trotting speed (i.e., trot-gallop transition speed) occurs at relatively faster speeds for larger quadrupeds. The gait referred to as an amble, tölt, or “running walk” is another symmetrical gait used by quadrupedal mammals including lemurs, monkeys, elephants, and some horses (reviewed by Schmitt et al., 2006). It is a four-beat gait with phase relationships like those of walking but with speeds comparable to fast trotting. The amble reduces vertical oscillations of the center of mass and this relatively flat trajectory is thought to be advantageous for locomotion on branches (e.g., Cartmill et al., 2002) and as a fast gait that allows very large quadrupeds to avoid aerial periods (e.g., Hutchinson et al., 2003).

At intermediate to high speeds, quadrupeds use asymmetrical gaits, such as the gallop, half-bound, bound, and pronk. Sprawling quadrupeds like salamanders and lizards do not use asymmetrical gaits, although juvenile crocodiles are able to gallop with an upright posture (Zug, 1974; Renous et al., 2002). Asymmetrical quadrupedal gaits are linked with upright limb posture, where legs are retracted in the parasagittal plane. Because left-right pairs of fore- and hindlimbs may be kept more nearly in phase, asymmetrical gaits allow bending of the spine and pelvis to contribute to stride length, with the muscles of the trunk used to bilaterally dorsiflex and ventroflex the spine. The left-right phase separation during galloping is typically 10–35% in the forelimb pair and 10–25% in the hindlimb pair (Afelt et al., 1983; Alexander, 1984). During bounding, both left-right pairs are in phase, and, during half-bounding, the hind left-right pair are in phase while the fore left-right pair are somewhat out of phase. Deer, antelope, goats, and sheep use the bound and pronk for display or warning and in steep terrain. The pronk is a pronounced bouncing gait where all four feet strike the ground in unison. The half-bound is well-known in rabbits and is the fast gait most frequently used by rodents and other small mammals. Rodents tend to use the half-bound at speeds corresponding to the upper trotting range of larger quadrupeds.

The gallop is the most commonly used asymmetrical gait of quadrupeds larger than about five kilograms. Broadly defined, it includes loping or cantering—a slow, three-beat gait with one diagonal pair in-phase, being preceded by a single hindlimb and followed by a single forelimb. Wolves use the lope alternately with trotting when traveling long distances. Large mammals, that have longer forelimbs and a forward center of mass, such as hyenas and bison, have a reduced trotting range and switch to a lope at intermediate speeds where other quadrupeds would trot. The transverse gallop is a faster four-beat gait, introducing a phase separation in the lope's diagonal pair with the pair's forefoot following the hind foot. The rotary gallop is the fastest gait used by quadrupedal mammals and it changes the order of the forefoot contacts such that the contact sequence is circular rather than crossing in a figure-eight. Galloping typically has a single gathered suspension period but cheetahs and grayhounds add an extended suspension period between hind- and forelimb contacts that serves to increase stride length at the fastest speeds.

### Speed effects

Animals choose a gait depending on their speed—they choose to walk at slow speeds and typically transition to symmetrical running or trotting at intermediate speeds and then asymmetrical half-bounding or galloping at fast speeds. The choice of symmetrical or asymmetrical gaits at faster speeds is related to leg posture (sprawling vs. upright), body mass, body mass distribution, rotational inertia of the body and appendages, leg length relative to body length, and fore-hind leg length differences. Gait choice and relative speeds at gait transitions may also change during uphill or downhill locomotion, or with varying substrate properties, or with uneven conditions in rugged terrain.

The analysis of phase relationships is generally effective for identifying and classifying gait, yet bipedal walking cannot be distinguished from running and quadrupedal walking from ambling based on foot sequencing alone. Thus, as introduced above, duty factor is typically used to distinguish these slower walking gaits from running and ambling. Greater duty factors are expected at slower speeds, and this provides a convenient method to discern gait differences using only temporal parameters. Despite its utility in gait analysis, duty factor cannot necessarily distinguish differences in gait dynamics. For example, walking and grounded running in birds have equal phase relationships and both have duty factors >0.5. Because duty factor decreases with speed, it can be associated with different gaits, as in the separation of walking from running of humans. Nonetheless, duty factor and footfall sequencing do not directly determine the dynamics underlying a particular gait and often lead to conflicting definitions of gait.

## Center of mass dynamics

Identifying the temporal pattern of leg sequencing, as measured by relative phase and duty factor, is a critical first step in quantifying gait, yet it is often imprecise and is insufficient to reveal the physical basis of locomotion. The dynamics of a given gait can only be known by measuring the forces determining the motion of the center of mass (CoM). This section first describes the measurement of CoM dynamics to determine oscillations resulting from combined leg force. It then describes two conventional models of gait: the rigid inverted pendulum (RIP) model traditionally applied to walking; and the spring-loaded inverted pendulum (SLIP) model for running. We conclude the section with a discussion of mechanical cost analysis that evaluates CoM dynamics more objectively, without the *a priori* assumptions required by the RIP and SLIP models.

### CoM measurements and gait patterns

Two approaches commonly used in the analysis of gait are a point mass model and a rigid-body model. The most basic measurement of gait dynamics treats the center of mass as a point mass, thus neglecting rotations about the center of mass (CoM). A point mass model of locomotion requires that forces exerted on all of the legs be summed and the instantaneous accelerations of the center of mass be determined throughout a complete stride (Cavagna, 1975). In contrast, a rigid-body model, requires separate measurements of forces exerted on each of the legs, as well as any torques, in order to consider not only the translation of, but also rotations about the center of mass. When more than one leg contacts the ground, as in the example of the diagonal limb pair during trotting, differential forces can produce force couples or “free moments,” which act about the center of mass to resist rotation and contribute to balance (Gray, 1968; Murphy and Raibert, 1985; Lee et al., 1999). Torques exerted at the feet also influence rotation in a rigid body model but these are typically restricted to the yaw axis, unless the foot can adhere to or grasp the substrate. Here we focus on evaluating the dynamics of a point mass model of the center of mass, emphasizing the principal gaits of bipeds and quadrupeds.

During legged locomotion, forward progression is coupled with vertical oscillations of the CoM. This forward progression is achieved either by using one leg at a time, as during bipedal running with aerial phases, or by using more than one leg at a time, as during all other gaits of bipeds, quadrupeds, and multilegged animals. The net effect of the vertical and shear ground reaction forces summed across all legs, less the vertical force of body weight, is a cyclic redirection of the CoM. Hence, the summed vertical force always oscillates about body weight with equal variation above and below—otherwise, the CoM would have a net rise or fall during a stride. The summed fore-aft forces exerted on the legs oscillate about zero with equal propulsive and braking impulse during steady-speed locomotion. At the beginning of stance, limbs are placed forward (protracted stance)—exerting force against the direction of travel, and at the end of stance, limbs are placed backward (retracted position)—exerting force in the direction of travel. Thus, legs typically exert braking followed by propulsive force (Gray, 1968). When more than one leg is in contact with the ground, the summed vertical forces and the summed shear forces determine oscillations of the center or mass. The pattern of combined forces acting on the center of mass is influenced by the sequencing and duration of leg contacts—explaining in many cases the correlation of relative phase and duty factor with the dynamics of the center of mass.

For a given gait, the CoM will either oscillate once or twice per stride. Symmetrical gaits, such as bipedal walking and running, have two vertical oscillations per stride. These oscillations are achieved alternately by left and right legs of a pair, and each of these legs contributes to just one oscillation as long as each leg's duty factor is <0.5. During asymmetrical gaits, such as bipedal hopping and quadrupedal galloping, there is only one vertical oscillation of the CoM per stride. The legs of right-left pairs act either in unison or in a staggered sequence to achieve this single cycle of oscillation and an aerial phase separates the leg contacts of one stride from those of the next (Hildebrand, 1980). Exceptions to the rule of a single aerial phase include the fast gallop of grayhounds and cheetahs, as well as the fast half-bound of hares (Hildebrand, 1980, Bertram and Gutmann, 2008), which add a second aerial phase—an “extended suspension” between hind- and foreleg contacts—while preserving the typical “gathered suspension” between fore- and hind-leg contacts. Barring these exceptions, asymmetrical gaits include one vertical oscillation per stride and symmetrical gaits include two vertical oscillations per stride.

**Figure 2:**
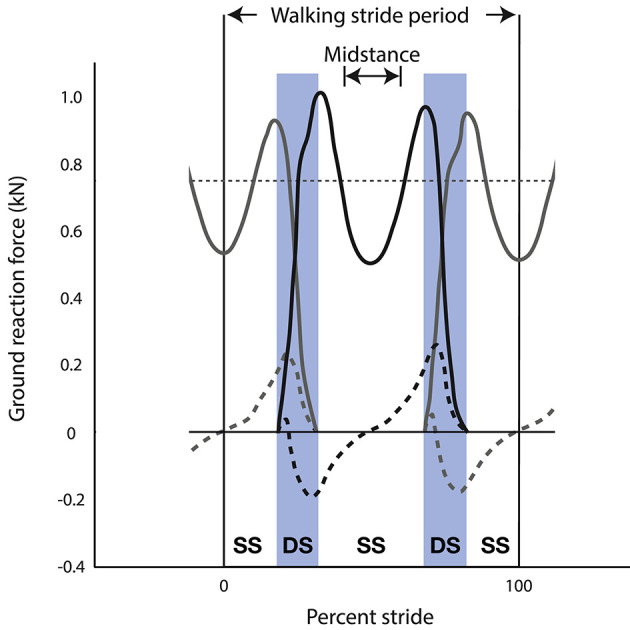
Ground reaction forces of the left limb (gray) and right limb (black) during three steps of human walking (i.e., left-right-left)—showing vertical (solid lines) and fore-aft (dashed lines) components of force. This stride is defined from mid-stance of the left limb to the subsequent mid-stance of the left limb. Vertical ground reaction force (solid lines) is below body weight during mid-stance of single stance (SS) and peaks at values greater than body weight near the beginning and end of double stance (DS). Fore-aft ground reaction force (dashed lines) peaks during double stance (DS) for both positive (propulsive) force from the trailing limb and negative (braking) force from the leading limb.

As already emphasized, total vertical force rises cyclically above and below body weight during the stride to achieve vertical oscillations of the CoM. This is true of both running and walking, although during bipedal walking the rise above body weight is achieved by simultaneous leg contacts during double support of the step-to-step transition (Figure 2), whereas a single leg contact achieves the rise above body weight during bipedal running. As a result of these differences between running and walking, vertical acceleration is downward during mid-stance of walking (Figure 2) and upward during mid-stance of running. Because acceleration is one-half cycle out of phase with its second derivative, position, the CoM reaches its lowest point during mid-stance of running and its highest point during mid-stance of walking. This traditional observation of the vertical position of the CoM at mid-stance is the basis for a long-standing dichotomy between walking and running (Cavagna et al., 1976, 1977).

### SLIP, RIP, and BSLIP models of locomotion

Running and walking have been characterized separately based on the phase relationships of their kinetic and potential energy oscillations which are typified by the spring-loaded inverted pendulum (SLIP) model and the rigid inverted pendulum (RIP) model. The view of kinetic and potential energy oscillations being either in phase, implying a SLIP model, or out of phase, implying a RIP model, remains the most influential construct in the field of gait analysis, including that of bipedal, quadrupedal, and multi-legged locomotion (reviewed by Dickinson et al., 2000). As explained in the previous section, patterns of vertical force dictate that the CoM reaches its lowest point (*minimum* potential energy) in mid-stance of running and its highest point (*maximum* potential energy) in mid-stance of walking. Despite this difference in potential energy at mid-stance, fluctuations in kinetic energy are determined primarily by changes in forward velocity, which follow the same pattern for running and walking. This is because braking occurs in the first half of a given leg's stance and propulsion in the second half. Thus, CoM velocity—and therefore kinetic energy—tends to reach a *minimum* near mid-stance in both walking and running.

In the case of running or other SLIP-like gaits, kinetic and potential energy both reach a *minimum* at mid stance—making these energies more-or-less in phase. SLIP-like gaits include bipedal running and hopping, as well as quadrupedal and multilegged trotting, which are well-described as “bouncing” gaits because the greatest leg compression occurs at about the same time as the greatest vertical force (Blickhan, 1989; McMahon and Cheng, 1990). If physical leg springs are present, these spring-like dynamics provide some opportunity for energy savings by elastic storage and return of some portion of the absorptive and generative work done by muscles or robotic actuators. Current research hybridizes SLIP concepts with rigid body models and link-segment models (reviewed by Sharbafi and Seyfarth, 2017). For example, mass distribution away from the hip influences dynamics of the spring-loaded leg in a bipedal running model (Blickhan et al., 2015). This simulated biped is controlled using the virtual pivot point construct to direct the resultant force vector through a point above the CoM (Maus et al., 2008; Sharbafi and Seyfarth, 2017).

In contrast to SLIP-like gaits, potential energy tends to reach a *maximum* near mid-stance during walking—making kinetic and potential energies more-or-less out of phase. According to the conventional interpretation of “two basic mechanisms,” this is sufficient to invoke a RIP model of walking dynamics. Nonetheless, experimental studies show that bipedal and quadrupedal walking dynamics (e.g., Lee and Farley, 1998; Griffin et al., 2004, Genin et al., 2010) do not match the RIP model. This is unsurprising given that an actual rigid inverted pendulum (i.e., a mass on a massless rod of fixed length) would show a peak vertical force instead of a minimum vertical force in the mid-stance position, as described by Geyer et al. (2006). The same authors propose an alternative compliant-legged model of walking called the bipedal SLIP or BSLIP, which reproduces the characteristic m-shaped force with a minimum at mid-stance by providing a spring-loaded leg that introduces compliance. The BSLIP also includes summation of leading and trailing leg forces during double support of the step-to-step transition. Although this revealing model is widely used and highly cited, the BSLIP has yet to upend the RIP model of walking in most textbook explanations. This may be partly because the BSLIP is more challenging to simulate and perhaps also because its conservative leg springs limit its ability to match the full range of human walking speeds (Lipfert et al., 2012). Nonetheless, a recent bipedal robot demonstrates SLIP-like running and BSLIP-like walking, using the same spring-loaded legs (Hubicki et al., 2018). In principle, the BSLIP model is more correct than the unrealistic impulsive step-to-step transition of the RIP model because it captures the m-shaped force profile and allows for double support.

### Mechanical cost analysis

Instead of focusing on phase relationships between kinetic and potential energies, mechanical cost analysis quantifies the mechanical cost of redirecting an animal's center of mass and reveals the underlying physics of that cost. This approach considers each instance of the stride. MCA reduces the observed center of mass dynamics to dimensionless parameters—the key amongst these being collision angle, which is equivalent to mechanical cost of transport (Lee et al., 2011). Using this approach, gaits are not constrained to *a priori* models that often invoke idealized gaits as ill-fitting approximations. Instead, mechanical cost analysis applies across gaits and species by focusing on the fundamental physics of the animal's interaction with the substrate. MCA provides further insight into not only the overall stride dynamics but also the individual events in the stride, indicating which phases of the stride are more (or less) costly.

**Figure 3:**
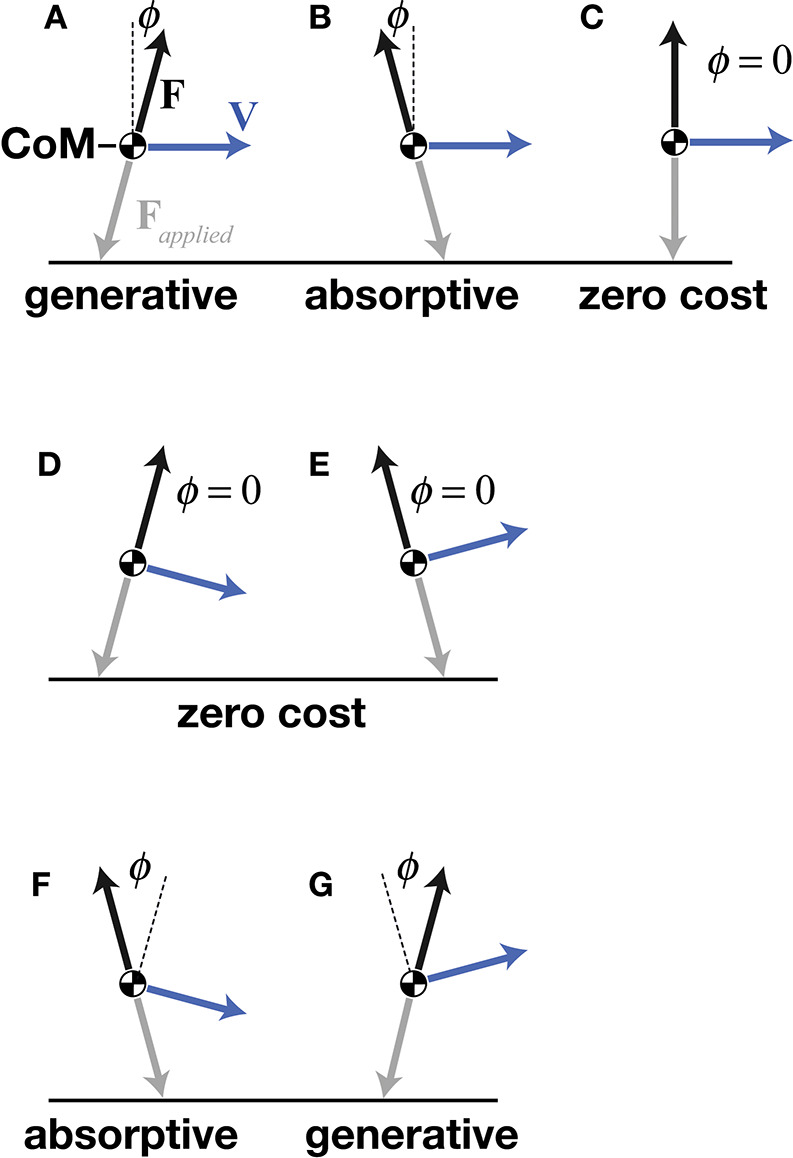
A point mass model of the center of mass (CoM), showing a simple case of a purely horizontal velocity vector **V** with propulsive **(A)**, braking **(B)**, and purely vertical **(C)** ground reaction force vectors **F**. Deviation of collision angle ϕ in the direction of **V** indicates generative cost and deviation of ϕ in the opposite direction of **V** indicates absorptive cost. Orthogonal constraint **(C)** incurs no mechanical cost. When **V** has a downward vertical component, orthogonal constraint is achieved by a propulsive **F (D)**, and when **V** has an upward vertical component, orthogonal constraint is achieved by a braking **F (E)**—both resulting in redirection of the CoM with zero mechanical cost. SLIP-like redirection of the CoM incurs high mechanical costs because **V** is downward during braking **(F)** and upward during propulsion **(G)**.

The central concept of mechanical cost analysis is d'Alembert's (1743) “principle of orthogonal constraint,” which shows that a mass can be redirected without mechanical work, as long as the constraint (force vector) is perpendicular to the path (velocity vector), such that their dot-product (mechanical power) is zero (Figures 3D,E). Considering the simple case of a flat trajectory, where the velocity vector is purely horizontal; the velocity of the center of mass would be increased by a forward-directed (propulsive) force vector or, decreased by a backward-directed (braking) force vector—these two conditions require generative or absorptive work, respectively, both incurring mechanical cost (Figures 3A,B). In contrast, a force vector (to resist gravity) does not change the velocity of the center of mass, thus requiring no work and moving with zero mechanical cost of transport (Figure 3C). In the latter example, the force and velocity vectors are kept perpendicular, matching the mechanics of an idealized wheel rolling on a flat surface. Although legged locomotion necessarily includes vertical oscillations, the benefit of moving without mechanical cost could be had as long as force and velocity vectors could be kept perpendicular throughout the vertical oscillations of the stride.

d'Alambert's theoretical redirection with zero work is not completely realized by legged systems because the ability to exert orthogonal forces is limited by a leg's position with respect to the CoM, its kinematic range of motion, and its force-torque capacity, amongst other factors. Nonetheless, certain gaits of bipeds, quadrupeds, and brachiators approximate orthogonal constraint. Deviations from orthogonal constraint require work. Specifically, work is required in any instance where force and velocity vectors deviate from a perpendicular relationship and the resulting mechanical power is non-zero (Ruina et al., 2005; Lee et al., 2011). Foundational studies in this area used mathematical models of discrete leg contacts to estimate the mechanical cost of redirecting the CoM for an entire stride of a given gait while considering leg number, step-to-step transition, or foot shape (McGeer, 1990; Kuo, 2001; Ruina et al., 2005). Mechanical cost analysis extends these previous models and captures the dynamics of the CoM not only for the entire stride but also at each instance of the stride by measuring the CoM velocity and ground reaction force on the CoM and then determining the instantaneous angle between these vectors. This analysis applies to both simulated models and experimental measurements.

Experimental data using mechanical cost analysis show that mechanical cost of transport is greater during running than during walking. Walking reduces mechanical cost by maintaining a more perpendicular relationship between force and velocity vectors throughout the stride. For example, during the step-to-step transition of human walking, also called double stance, the CoM (and thus, velocity) transitions from downward to upward while ground reaction force transitions from propulsive (forward) to braking (backward). Specifically during double stance, the force is first propulsive (dominated by the trailing leg) and then braking (dominated by the leading leg). In this way, force and velocity vectors are kept more nearly perpendicular during downward to upward redirection of the center of mass.

In contrast, SLIP-like gaits, such as running, cannot approach orthogonal constraint because they instead follow a braking-propulsive pattern that simply aligns with the orientation of the support leg(s). Because bipedal running typically has only single-leg support, it cannot employ the propulsive and then braking pattern of the walking double stance. Instead, the SLIP-like pattern of a single leg's stance starts with braking (backward-directed force vector) with a large projection on the downward-directed velocity vector, followed by propulsion (forward-directed force vector) with a large projection on the upward-directed velocity vector (Figures 3F,G). In the SLIP construct, both early and late stance geometries deviate from orthogonal constraint, resulting in greater work during the downward to upward redirection of the CoM.

Mechanical cost analysis quantifies mechanical cost by measuring the collision angle ϕ, which is the deviation from perpendicular of the CoM force and velocity vectors. This angle is measured in each instance of the stride and is given by Lee et al. (2011):

(1) $$\begin{array}{c} \phi =\arcsin\left(\left|\text{F}\cdot \text{V}\right|\text{/}\left|\text{F}\|\text{V}\right|\;\right) \end{array}$$

where F is the force vector and V is the velocity vector, either in two or three dimensions. The overall collision angle across the entire stride Φ is a weighted-average of ϕ, where the weights are the magnitudes of force and velocity vectors in each instance:

(2) $$\begin{array}{c} \Phi \sum \left|\text{F}\|\text{V}\right|\phi \text{/}\sum \left|\text{F}\|\text{V}\right|\; \end{array}$$

Substituting the small angle approximation of Equation (1) into Equation (2) then shows that stride collision angle Φ is approximately equal to the mechanical cost of transport:

(3) $$\begin{array}{c} CoT_{mech}=\sum \left|\text{F}\cdot \text{V}\right|/n\bar{V_{y}}mg\;\equiv \sum \left|\text{F}\cdot \text{V}\right|\text{/}\sum \left|\text{F}\|\text{V}\right|\; \end{array}$$

where m is body mass, g is gravitational acceleration, $\bar{V_{y}}$ is the mean forward velocity, and *n* is the number of sampled points in a given stride. Mechanical cost of transport was originally derived using dimensional analysis and it gives the work to move a unit body weight a unit distance during steady-speed locomotion. However, only mechanical cost analysis reveals the physical basis for this cost. Specifically, the collision angle provides a physical basis for the mechanical cost of transport as well as a way to quantify the cost of individual events or phases within the stride. In contrast, CoT_mech_, in its original form, depends upon average forward velocity and only applies to a full stride of locomotion, without providing information about the costs of individual events or phases.

**Figure 4:**
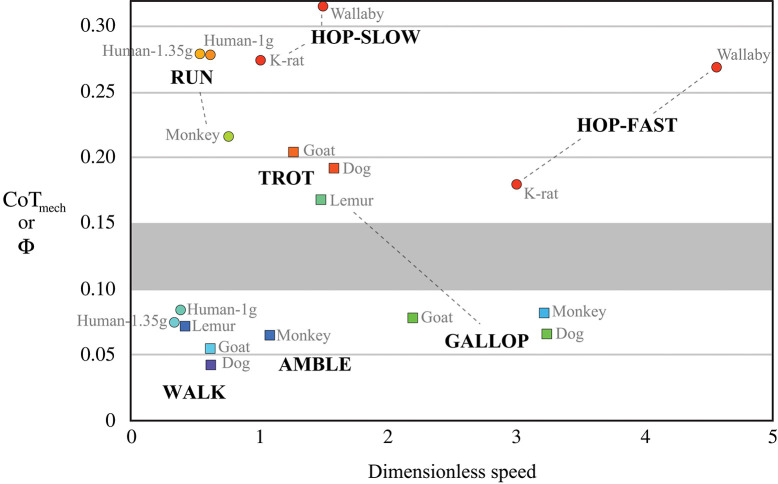
Mechanical cost of transport CoT_mech_ as a function of dimensionless speed in bipedal and quadrupedal mammals (reviewed by Lee et al., 2013). Cool colors represent gaits that target orthogonal constraint as a strategy to reduce CoT_mech_, which is equivalent to stride collision angleΦ. Warm colors represent SLIP-like “bouncing” gaits, having two- to five-times greater CoT_mech_ at a given dimensionless speed. An apparent boundary zone (gray shading) suggests a discrete separation between SLIP-like gaits and those targeting orthogonal constraint.

From the point mass perspective, SLIP-like bouncing gaits incur greater mechanical cost of transport—i.e., require more mechanical work to move the body weight a unit distance—than walking gaits that reduce required work by targeting orthogonal constraint. In humans, for example, the mechanical cost of transport during SLIP-like running is three-times that of walking (Figure 4, compiled by Lee et al., 2013). Again, this lower mechanical cost of walking is achieved by summed forces on the trailing and leading legs during double support, providing propulsion while the velocity is directed downward, then braking while the velocity is directed upward. This is the opposite pattern of SLIP-like gaits, such as running, and it allows human walking dynamics to more nearly approach orthogonal constraint, thereby reducing required work and achieving oscillations of the CoM with reduced mechanical cost.

Work is also mitigated by brachiating siamangs (Michilsens et al., 2012) in the same way but to an even greater extent than in terrestrial walking. Instead of negotiating substrate interactions with one or more foot, as discussed so far, these apes use the overhead support of branches to achieve fast, smooth locomotion through the canopy. Brachiators are able to reduce required work using a single handhold because they can either pull or push on the handhold to keep force and velocity vectors more perpendicular, whereas terrestrial animals can only load their legs in compression. Hence, brachiating animals can achieve propulsion while their velocity is directed downward and braking while their velocity is directed upward—i.e., the same strategy of orthogonal constraint that is approached using two legs during the step-to-step transition of human walking or by four legs during quadrupedal galloping (Bertram and Gutmann, 2008; Lee et al., 2011). The astonishingly smooth movement of brachiating gibbons, in fact, helped initiate a resurgence of theoretical studies of collision mechanics, which model generative and absorptive events during locomotion (Usherwood and Bertram, 2003; Ruina et al., 2005; Bertram, 2016). While suspensory locomotion and inverted quadrupedal walking generally show a propulsive-then-braking pattern, (Ishida et al., 1990; Chang et al., 2000; Granatosky, 2015; Granatosky et al., 2016; Granatosky and Schmitt, 2017), mechanical cost analysis has yet to be applied to these data.

Mechanical cost analysis of animals can also be evaluated in light of theoretical predictions of how work can be mitigated by legged systems. A key finding of this mathematical approach is that mechanical cost of transport is reduced in proportion to the number of contacts used to achieve a given redirection of the CoM (Ruina et al., 2005). In other words, the mechanical cost of redirecting the center of mass is theorized to be divided by the number of sequenced leg contacts. For canine walking, as compared with human walking (see leg contact sequences in Figure 1), this prediction is borne out by mechanical cost analysis of experimental data, which shows about one-half the mechanical cost incurred for four sequenced leg contacts of a dog as for two sequenced leg contacts of a human (Figure 4). The gallop and amble are faster quadrupedal gaits that use sequenced leg contacts to reduce mechanical cost of transport but not quite to the extent of quadrupedal walking (Figure 4). The concept of reducing cost by increasing the number of sequenced ground contacts has been extended to human walking by modeling contacts of the heel and toe as discrete “leg” contacts (Ruina et al., 2005). Just as for sequenced contacts of separate legs, this model predicts that sequenced heel and toe contacts halve the mechanical cost of transport during walking.

Another important theoretical prediction is that work can be reduced by increasing the length of the foot. Simulations show that a foot that extends forward the full length of the step and that is just slightly rounded (convex relative to the ground), as opposed to cupped (concave), achieves work-free redirections from one leg contact to the next during walking—this case is likened to a polygon with convex surfaces rolling smoothly between pendular support phases at its vertices (Ruina et al., 2005). However, effects of the foot's length and its radius of curvature cannot be easily separated because, during single-leg stance, a rounded foot centered on a rigid leg prescribes the path of the center of mass in addition to lengthening the foot in both the “toe” and “heel” directions. The question of foot shape has been addressed in passive dynamic walking robots and simulations (e.g., McGeer, 1990; Kuo, 2001), as well as in human studies with experimental boots of different lengths and radii (Adamczyk et al., 2006). In the latter experiment, mechanical cost was reduced by simultaneously increasing the length and radius of curvature, a result that is similar to theoretical predictions based on foot length alone (Ruina et al., 2005).

It stands to reason that hexapedal and octopedal animals would have even lower mechanical cost than quadrupeds by using more than four sequenced leg contacts, however, mechanical cost analysis has not yet been applied to arthropods. The ideal of orthogonal constraint could be approached more nearly by eight sequenced leg contacts; however, arthropods primarily use alternating tripods to achieve SLIP-like trotting gaits (reviewed by Holmes et al., 2006). It is plausible that work reduction might not be as strongly selected in small arthropods—perhaps due to scaling of energetics, terrain interactions, or constraints on motor control. Whatever the explanation, mechanical cost analysis holds potential to further explore nature's solutions to multi-legged gait.

### Comparing legged animals and machines

Legged robots designed for economy are typically slower than animals of similar size (e.g., Collins et al., 2005), with a few exceptions, such as the MIT Cheetah quadruped, which uses regenerative motors (Seok et al., 2015). Humans walk slowly (1.0 ms^−1^) with a mechanical cost of transport of ~0.05 and a total cost of transport of ~0.41 (Voloshina et al., 2013), so mechanical cost represents about one-eighth of the total cost. If we assume no co-contraction between antagonist muscle pairs and a muscle efficiency of 25%, the muscle energy needed for CoM oscillations would represent just one-half of the total cost of transport. Hence, the remaining half must be spent on muscle energy needed for co-contraction, work of the legs against one another, and work for swinging of the arms and legs. In comparison to humans, the world's most economical bipedal robot, Cornell Ranger, achieves a mechanical cost of transport of ~0.08 (determined by doubling the cost of positive motor work) at a speed of 0.6 ms^−1^ (Bhounsule et al., 2014). After subtracting the energy used by its control system, Cornell Ranger has a total (electrical) cost of transport ~0.11, and all of this energy is accounted for by positive and negative motor work with an average motor efficiency of 65%. Walking at a little more than one-half the speed of a human walking slowly, Cornell Ranger would need improved dynamics to both reach typical human walking speeds and achieve a mechanical cost of transport as low as 0.08.

### Summary

Legged gaits were first identified by the relative phases of leg contacts, which also identifies the sequence of leg contacts. A gait is defined as symmetrical when the relative phases of corresponding left-right pairs are separated by one-half stride cycle, such as in a striding biped or a trotting quadruped. When a left-right pair is used in unison (such as in hopping) or has staggered contacts (as in quadrupedal galloping), the gait is considered asymmetrical.

Locomotion is more correctly evaluated by measuring the dynamics of the center of mass (CoM). Traditional models have compared the phases of the CoM's potential and kinetic energy, using a spring-loaded inverted pendulum (SLIP) model when these energies are relatively in phase and a rigid inverted pendulum (RIP) model when they are out of phase. More recent work has introduced mechanical cost analysis that quantifies the work a gait requires by using the geometry of force and velocity vectors of the CoM.

All legged gaits include cyclic oscillations of the CoM; symmetrical gaits use two vertical oscillations, and asymmetrical gaits typically consist of only a single vertical oscillation per stride. SLIP-like “bouncing” gaits may be symmetrical (e.g., running and trotting) or asymmetrical (e.g., hopping and bounding), but they always couple braking with downward vertical velocity and propulsion with upward vertical velocity, resulting in mechanical work performed by the animal. Other symmetrical (e.g., walking and ambling) and asymmetrical (e.g., galloping and half-bounding) gaits, however, couple propulsion with downward vertical velocity and braking with upward vertical velocity, reducing an animal's mechanical cost of transport. This latter pattern takes advantage of d'Alembert's principle of orthogonal constraint by keeping force and velocity vectors more nearly perpendicular in each instance of the stride—thus reducing the mechanical cost of transport, which is zero in the idealized case of orthogonal constraint. Slow symmetrical walking gaits and faster asymmetrical galloping gaits exploit this principle to reduce work, and thus reduce the mechanical cost of transport. This reduction of mechanical cost of transport is a key feature of several gaits including bipedal and quadrupedal walking, fast gaits with multiple sequenced leg contacts, and brachiation. Theoretical mechanics show that increasing the number of sequenced leg contacts, dividing single foot contacts into multiple contacts, and increasing foot length are all mechanisms that can reduce mechanical cost of transport, as measured by mechanical cost analysis. Together with an increasing body of data from comparative animal locomotion, these principles hold substantial promise for the design and control of legged robots and prostheses that can achieve economical locomotion by reducing the mechanical cost.

## Author contributions

DL wrote approximately 60% of this review paper. SH wrote approximately 40% of this review paper.

### Conflict of interest statement

The authors declare that the research was conducted in the absence of any commercial or financial relationships that could be construed as a potential conflict of interest.
